# Progress Toward Tuberculosis Elimination and Tuberculosis Program Performance — National Tuberculosis Indicators Project, 2016–2022

**DOI:** 10.15585/mmwr.ss7304a1

**Published:** 2024-06-06

**Authors:** Rachel Woodruff, Robert Pratt, Maureen Kolasa

**Affiliations:** 1 Division of Tuberculosis Elimination, National Center for HIV, Viral Hepatitis, STD, and TB Prevention, CDC

## Abstract

**Problem/Condition:**

Elimination of tuberculosis (TB) is defined as reducing TB disease incidence in the United States to less than 1 case per million persons per year. In 2022, TB incidence in the United States was 2.5 TB cases per 100,000 persons. CDC’s TB program developed a set of national TB indicators to evaluate progress toward TB elimination through monitoring performance of state and city TB program activities. Examining TB indicator data enables state- and city-level TB programs to identify areas for program evaluation and improvement activities. These data also help CDC identify states and cities that might benefit from technical assistance.

**Period Covered:**

The 5-year period for which the most recent data were available for each of five indicators: 1) overall TB incidence (2018–2022), 2) TB incidence among non–U.S.-born persons (2018–2022), 3) percentage of persons with drug susceptibility results reported (2018–2022), 4) percentage of contacts to sputum acid-fast bacillus (AFB) smear-positive TB patients with newly diagnosed latent TB infection (LTBI) who completed treatment (2017–2021), and 5) percentage of patients with completion of TB therapy within 12 months (2016–2020).

**Description of System:**

The National TB Indicators Project (NTIP) is a web-based performance monitoring tool that uses national TB surveillance data reported through the National TB Surveillance System and the Aggregate Reports for TB Program Evaluation. NTIP was developed to facilitate the use of existing data to help TB program staff members prioritize activities, monitor progress, and focus program improvement efforts. The following five indicators were selected for this report because of their importance in Federal TB funding allocation and in accelerating the decline in TB cases: 1) overall TB incidence in the United States, 2) TB incidence among non–U.S.-born persons, 3) percentage of persons with drug susceptibility results reported, 4) percentage of contacts to sputum AFB smear-positive TB cases who completed treatment for LTBI, and 5) percentage of patients with completion of TB therapy within 12 months. For this report, 52 TB programs (50 states, the District of Columbia, and New York City) were categorized into terciles based on the 5-year average number of TB cases reported to National TB Surveillance System. This grouping allows comparison of TB programs that have similar numbers of TB cases and allocates a similar number of TB programs to each category. The following formula was used to calculate the relative change by TB program for each indicator: [(% from year 5 − % from year 1 ÷ % from year 1) × 100].

**Results:**

During the 5-year period for which the most recent data were available, most TB programs had improvements in reducing overall TB incidence (71.2%) and increasing the percentage of contacts receiving a diagnosis of LTBI who completed LTBI treatment (55.8%); the majority of programs (51.0%) also had improvements in reducing incidence among non–U.S.-born persons. The average percentage of persons with drug susceptibility results reported in most jurisdictions (28 of 52, [53.9%]) met or exceeded the 5-year national average of 97% (2018–2022). The percentage of contacts to sputum acid-fast bacillus (AFB) smear-positive TB patients with newly diagnosed latent TB infection (LTBI) who completed treatment increased in 29 of 52 (55.8%) jurisdictions from 2017 to 2021, signifying that, for most jurisdictions, steps have been taken to enhance performance in this area. The average percentage of patients with completion of TB therapy within 12 months was at or above the national average of 89.7% in approximately two-thirds (32 of 52 [61.5%]) of jurisdictions.

**Interpretation:**

This report is the first to describe a 5-year relative change for TB program performance. These results suggest that TB programs are making improvements in activities that help identify persons with TB and LTBI and ensure patients complete treatment in a timely manner.

**Public Health Action:**

Use of NTIP data from individual TB programs enables a more detailed examination of trends in program performance and identification of areas for program improvement. Assessing indicator trends by TB program provides an opportunity to gain a better understanding of program performance in comparison to other programs. It can also facilitate communication between programs regarding successes and challenges in program improvement. This information is valuable for TB programs to allocate resources effectively and provide additional context on TB control for public health policymakers.

## Introduction

Using data to monitor tuberculosis (TB) program performance can provide insights into program functioning and allow for the timely recognition of successes and areas for improvement (*1*,*2*). Monitoring and evaluating TB program activities involves a combination of objectives, indicators, and performance targets that allow for continuous tracking of program progress and performance monitoring. Performance indicators are specific, measurable criteria that are chosen based on national TB program objectives and are used to quantitatively assess progress and outcomes (*2*–*4*). Each national TB program indicator has an associated performance target. Together, these tools help programs assess the effectiveness of TB control activities and identify areas for improvement, thus improving TB patient outcomes (*5*,*6*).

CDC, in collaboration with internal and external partners, developed a set of national TB objectives, associated indicators, and performance targets to evaluate progress toward TB elimination by monitoring the performance of state and city TB program activities (*7*,*8*). The national TB indicators are available to TB programs through the National TB Indicators Project (NTIP) application (*8*).

CDC publishes annual TB surveillance reports (*9*), which presents national data over time and jurisdiction data for a single year, and state and city TB report (*10*), which display TB indicator data at a single point at the state and city levels. Neither of these reports provide trend data at the state, city, or local level. The findings in this report can be used by state and city TB programs to assess their TB control efforts and identify areas for improvement.

## Methods

To provide programs with a resource to evaluate their performance over time, CDC summarized NTIP data for five key indicators: 1) overall TB incidence in the United States, 2) TB incidence among non–U.S.-born persons, 3) percentage of persons with drug susceptibility results reported, 4) percentage of contacts to sputum acid-fast bacillus (AFB) smear-positive TB patients who received a new diagnosis of latent TB infection (LTBI) and who completed LTBI treatment (among those who started LTBI treatment), and 5) percentage of patients with completion of TB therapy within 12 months (among those who are eligible to complete within 12 months) (*9*). These indicators are included in a formula to determine the amount of funding provided to TB programs as part of the CDC TB Cooperative Agreement for the prevention and control of TB (*11*).

### Data Sources and Collection

NTIP is a web-based performance-monitoring tool that uses routine data reported to the National TB Surveillance System (NTSS) and the Aggregate Reports for Program Evaluation (ARPE). NTIP was developed to facilitate the use of NTSS data to measure national TB program objectives, indicators, and performance targets, which can help TB programs prioritize activities, monitor progress, and focus program improvement efforts. TB programs have access to their own specific data and national aggregate data in NTIP. However, they are unable to access performance data from other TB programs. As a result, they can only compare their program's performance trends with national averages.The ability to compare performance among TB programs that report similar numbers of TB cases could be more useful in identifying areas for improvement.

All 50 U.S. states, the District of Columbia, and New York City report each new case of TB to NTSS via the Report of Verified Case of TB (*9*). U.S. territories and freely associated states also report TB data to NTSS, but these data are not included in this report. Performance measures (rates and percentages) associated with TB incidence in the United States, TB incidence among non–U.S.-born persons, completion of TB therapy within 12 months, and drug susceptibility test results are calculated using NTSS data.

ARPE is a data collection system through which TB programs report information about persons who received a new diagnosis of TB disease or LTBI. ARPE information is submitted through NTIP using a standardized form (*12*,*13*). ARPE data are used to calculate the percentage of contacts who received a new diagnosis of LTBI and completed treatment.

The 52 TB programs included in this report correspond to distinct geographic jurisdictions. For the purposes of this report, the term jurisdiction will be used to describe results related to both the TB program and its corresponding geographic area.

### National TB Indicators and Performance Targets

The cohort for each national TB indicator is defined as the number of persons reported in the year of interest who are eligible to meet the performance objective for the indicator. For each indicator, inclusion and exclusion criteria are defined for the cohort. National performance targets for these indicators are calculated approximately every 5 years on the basis of historical TB program performance for each indicator (*14*) (Box).

BOX **Tuberculosis program indicators and 2025 national performance targets**

**Indicators and targets for reducing TB incidence**

- Incidence in the United States:* 1.3 cases per 100,000 persons
- Incidence among U.S.-born persons: 0.4 cases per 100,000 persons
- Incidence among non–U.S.-born persons:* 8.8 cases per 100,000 persons
- Incidence among U.S.-born persons who are non-Hispanic Black or African American: 1.0 cases per 100,000 persons
- Incidence among children aged <5 years: 0.1 cases per 100,000 children

**Indicators and targets for TB case management and treatment**

- Positive or negative HIV test result reported: 99%
- Treatment initiation within 7 days of specimen collection: 96%
- Began recommended initial 4-drug regimen: 97%
- Sputum culture result reported: 99%
- Documented conversion to negative results within 60 days of treatment initiation: 83%
- Completion of treatment within 12 months:* 95%

**Indicators and targets for laboratory reporting**

- Culture results reported by laboratory within 25 days of specimen collection: 78%
- Nucleic acid amplification test results reported by laboratory within 6 days of specimen collection: 97%
- Initial drug-susceptibility results reported:* 100%
- Genotyping results reported:* 100%

**Indicators and targets for contact investigations**

- TB patients who have contacts elicited: 100%
- Contacts who are examined for infection and disease: 94%
- Contacts with diagnosed latent TB infection who start treatment: 92%
- Contacts with diagnosed latent TB infection who complete treatment:* 93%

**Indicators and targets for examination of immigrants and refugees**

- Initiate a medical examination within 30 days of abnormal findings on chest radiograph notification: 72%
- Complete a medical examination within 120 days of abnormal findings on chest radiograph notification: 78%
- Immigrants and refugees with diagnosed latent TB infection or who have radiographic findings consistent with prior pulmonary TB who start treatment: 87%
- Immigrants and refugees with diagnosed latent TB infection or who have radiographic findings consistent with previous pulmonary TB who complete treatment: 87%

**Indicators and targets for data reporting**

- Completeness of each core Report of Verified Case of Tuberculosis data item reported to CDC: 100%
- Completeness of each core Aggregate Reports for Tuberculosis Program Evaluation data item reported to CDC: 100%
- Completeness of each core TB Follow-Up Worksheet data item reported to CDC via the Electronic Disease Notification System: 93%

**Indicators and targets for program evaluation**

- Submission of a program-specific performance monitoring plan and an annual program evaluation plan: 100%
- Designation of a TB training focal point: 100%

**Indicators and targets for human resource development**

- Submission of a program-specific human resource development plan and a yearly update of progress: 100%
- Designation of a TB training focal point: 100%

**Source:** 2025 National TB Program Objectives & Performance Targets (https://www.cdc.gov/tb/education/pdf/2025_TB_Objectives.pdf).

**Abbreviation:** TB = tuberculosis.

* Included in results.

The following indicators were selected for this report because of their importance in Federal TB funding allocation and in intensifying efforts to reduce TB incidence:

- TB incidence in the United States: number of new TB cases per 100,000 persons per year
- TB incidence among non–U.S.-born persons: number of TB cases among non–U.S.-born persons per 100,000 non–U.S.-born persons per year
- Drug susceptibility test results: percentage of persons with TB disease with a positive culture result who had initial drug-susceptibility results reported
- Contacts to sputum AFB smear-positive TB patients who received a new diagnosis of LTBI who completed treatment: percentage of contacts to persons with sputum AFB smear-positive TB who received a diagnosis of LTBI and who completed treatment among those patients who started treatment
- Completion of TB therapy within 12 months: percentage of patients who received a new diagnosis of TB disease for whom treatment completion within 12 months is indicated and who completed treatment within 12 months (366 days)

### Analysis

Descriptive analyses were performed for the five selected indicators for each of the 52 jurisdictions in the United States. For this report, jurisdictions were categorized into terciles based on the average number of cases during the 5-year reporting period for which the most recent data were available for the analyzed variables. This grouping allowed comparison of jurisdictions with similar numbers of TB cases and allocated an equivalent number of jurisdictions to each category.

The 5-year reporting period was 2018–2022 for overall TB incidence, TB incidence among non–U.S.-born persons, and persons with drug susceptibility results reported. For contacts who received a new diagnosis of LTBI who completed LTBI treatment, the 5-year reporting period was 2017–2021 because of different reporting deadlines associated with data from NTSS and ARPE. TB therapy can take up to 2 years to complete; therefore, 2016–2020 was the 5-year period for completion of TB therapy within 12 months.

The average percentage for each indicator over the 5-year period was calculated using the formula (% from Year 1 + % from Year 2 + % from Year 3 + % from Year 4 + % from Year 5) ÷ 5. The relative change from year 1 to year 5 for each indicator was calculated as [(% from Year 5 − % from Year 1) ÷ % from Year 1] × 100. For indicators based on incidence, the calculations used incidence rather than percentage for each year. The national average was calculated for each indicator (i.e., sum of average percentage or incidence over the 5-year period for all jurisdictions ÷ total number of jurisdictions). Data in the tables are displayed by improved relative change to allow TB programs to assess how they rank in relation to other jurisdictions with similar numbers of reported TB cases. Overall TB incidence and TB incidence among non–U.S.-born persons were calculated using 2015–2019 annual population estimates (the latest population data available) from the Census Bureau’s American Community Survey (*15*).

## Results

For 2018–2022, the terciles for overall TB incidence, TB incidence among non–U.S.-born persons, and percentage of persons with drug susceptibility results reported were jurisdictions with 1–50 TB cases (T1), 51–126 TB cases (T2), and 127 or more TB cases (T3) (Tables 1, 2 and 3). For 2017–2021, the terciles for the percentage of contacts with newly diagnosed LTBI who completed treatment were jurisdictions with 1–49 TB cases (T1), 50–130 TB cases (T2), or ≥131 TB cases (T3) (Table 4). For 2016–2020, the terciles for the percentage of patients with completion of TB therapy within 12 months were jurisdictions with 1–46 TB cases (T1), 47–130 TB cases (T2), or 131 or more TB cases (T3) (Table 5).

**TABLE 1:** Overall tuberculosis incidence,* by jurisdiction and reporting year — United States, 2018–2022^†,§^

Jurisdiction^¶,^**	5-year annual average number of cases^††^	2018 incidence*	2019 incidence*	2020 incidence*	2021 incidence*	2022 incidence*	5-year annual average incidence*^,§§^	Relative change in incidence* from 2018 to 2022 (%)^¶¶^
**5-year average of ≥127 cases**								
Maryland	183	3.4	3.5	2.5	3.1	2.5	3.0	−26.0
Minnesota	141	3.1	2.6	2.1	2.3	2.3	2.5	−24.5
Massachusetts	165	2.9	2.6	2.1	2.2	2.2	2.4	−24.4
Pennsylvania	181	1.7	1.5	1.2	1.3	1.3	1.4	−19.4
Ohio	151	1.5	1.3	1.1	1.3	1.2	1.3	−18.6
North Carolina	176	1.9	1.8	1.5	1.7	1.5	1.7	−18.2
Arizona	156	2.5	2.5	1.9	1.8	2.1	2.1	−14.7
Florida	519	2.8	2.6	1.9	2.3	2.5	2.4	−11.5
California	1,901	5.3	5.3	4.3	4.5	4.7	4.8	−11.1
New York	174	1.7	1.7	1.4	1.4	1.6	1.6	−8.6
Illinois	283	2.5	2.6	1.7	2.0	2.4	2.2	−6.1
Virginia	184	2.4	2.2	2.0	1.9	2.3	2.1	−5.8
Georgia	255	2.6	2.8	2.1	2.1	2.4	2.4	−5.5
New Jersey	284	3.3	3.5	2.8	3.1	3.1	3.1	−4.2
Texas	1,048	3.9	4.0	3.0	3.4	3.7	3.6	−4.2
New York City	524	6.6	6.7	5.3	6.2	6.3	6.2	−3.9
Washington	205	2.5	2.9	2.1	2.6	3.2	2.7	28.6
**5-year average of 51–126 cases**								
Mississippi	56	2.7	1.9	1.4	1.6	1.8	1.9	−31.7
Alabama	81	1.8	1.8	1.5	1.8	1.3	1.6	−30.0
Tennessee	114	2.1	1.9	1.7	1.2	1.5	1.7	−26.0
Hawaii	104	8.4	7.0	6.5	7.4	7.0	7.3	−17.1
Indiana	108	1.7	1.6	1.4	1.9	1.5	1.6	−16.1
Missouri	75	1.3	1.1	1.3	1.2	1.1	1.2	−14.3
Nevada	60	2.3	1.7	1.9	1.9	2.0	2.0	−13.3
Colorado	59	1.1	1.1	0.9	1.0	1.0	1.0	−12.7
Oregon	74	1.9	1.7	1.6	1.9	1.7	1.8	−11.1
Arkansas	67	2.5	2.1	2.0	2.3	2.2	2.2	−10.9
Louisiana	95	2.3	1.9	2.1	1.9	2.1	2.0	-8.8
Wisconsin	51	0.8	0.9	0.6	1.1	0.9	0.9	4.6
Oklahoma	73	1.9	1.8	1.7	1.7	2.0	1.8	6.9
Kentucky	64	1.4	1.5	1.5	1.3	1.6	1.4	8.4
Michigan	119	1.1	1.3	1.0	1.4	1.2	1.2	10.5
South Carolina	84	1.7	1.6	1.3	1.7	1.9	1.6	15.0
Connecticut	59	1.4	1.9	1.5	1.5	1.9	1.6	30.2
Alaska	66	8.5	7.9	7.9	7.9	13.0	9.1	51.8
**5-year average of 1–50 cases**								
District of Columbia	22	5.1	3.4	2.7	2.7	2.2	3.2	−56.3
Vermont	4	0.8	0.6	0.5	0.5	0.5	0.6	−41.8
Delaware	22	2.2	2.0	1.7	4.1	1.3	2.3	−40.3
Idaho	9	0.9	0.4	0.4	0.3	0.6	0.5	−32.3
North Dakota	13	1.7	2.4	1.3	1.9	1.3	1.7	−24.6
New Mexico	33	1.9	2.0	1.4	1.1	1.4	1.5	−23.8
South Dakota	13	1.4	1.8	1.8	1.3	1.1	1.5	−17.9
Rhode Island	15	1.8	1.3	0.7	1.6	1.6	1.4	−13.7
New Hampshire	11	0.9	0.4	0.9	0.9	0.8	0.8	−10.5
Wyoming	1	0.2	0.2	0.0	0.5	0.2	0.2	−0.2
Nebraska	26	1.4	0.9	1.7	1.1	1.5	1.3	5.5
Montana	4	0.5	0.2	0.4	0.3	0.5	0.4	15.4
Maine	16	1.0	1.3	1.3	1.0	1.2	1.2	18.4
Iowa	50	1.6	1.6	1.2	1.5	1.9	1.6	21.0
Utah	25	0.6	0.8	0.9	0.5	1.0	0.8	73.6
Kansas	39	1.0	1.3	1.3	1.5	1.8	1.3	84.3
West Virginia	9	0.3	0.5	0.7	0.3	0.6	0.5	85.7

**Abbreviation:** TB = tuberculosis.

* Number of TB cases per 100,000 persons. Population data from the Census Bureau’s American Community Survey, 2021. Jurisdictions were categorized into terciles based on the average number of cases during the 5-year reporting period for which the most recent data were available.

^†^ The 2025 national performance target is 1.3 cases per 100,000 population. The 2025 National TB Program Objectives & Performance Targets reflect the projected performance of the top 10% of TB programs in the United States (https://www.cdc.gov/tb/education/pdf/2025_TB_Objectives.pdf).

^§^ The 2018–2022 national average incidence was 2.5 cases per 100,000 population and was calculated as the 5-year sum of TB cases in all reporting areas divided by the 5-year sum of the population in all reporting areas and multiplied by 100,000 (i.e., [sum of cases reported 2018 through 2022 ÷ sum of population 2018 through 2022] × 100,000).

^¶^ Reporting areas (i.e., jurisdictions) include all states, New York City, and the District of Columbia. These areas are included because they report TB surveillance data directly to CDC. Reporting areas are categorized into three groups based on the average number of TB cases reported over the 5-year reporting period. The cutoffs for the groups were determined by the 5-year average number of cases that fell within the 33rd and 66th percentiles.

** Data for jurisdictions with <50 average cases should be interpreted with caution because of variability in performance results attributable to small denominators.

^††^ Calculated as the sum of cases reported over the 5-year period divided by 5 (i.e., [sum of cases reported 2018 through 2022] ÷ 5).

^§§^ Calculated as the sum of annual incidence divided by 5 (i.e., [sum of incidence reported 2018 through 2022] ÷ 5).

^¶¶^ Calculated as the difference in incidence between year 1 and year 5, divided by the incidence in year 1, multiplied by 100 (i.e., [(incidence in 2022 − incidence in 2018) ÷ incidence in 2018] × 100). Result is based on unrounded numbers.

**TABLE 2:** Tuberculosis incidence* among non–U.S.-born persons, by jurisdiction and reporting year — United States, 2018–2022^†,§^

Jurisdiction^¶,^**	5-year annual average number of cases^††^	2018	2019	2020	2021	2022	5-year annual average incidence among non-U.S.–born persons^§§^	Relative change from 2018 to 2022 (%)^¶¶^
**5-year average ≥127 cases**								
Minnesota	141	29.5	27.3	22.0	21.5	19.7	24.0	−33.0
Maryland	183	18.7	18.1	13.0	14.5	13.2	15.5	−29.5
Pennsylvania	181	17.0	16.8	13.4	12.9	13.2	14.6	−22.0
Massachusetts	165	14.4	12.5	10.5	11.0	11.4	12.0	−20.7
Ohio	151	23.3	17.5	16.9	13.8	18.9	18.0	−19.2
Texas	1,048	14.0	14.6	11.2	12.2	11.4	12.7	−18.7
California	1,901	16.4	16.4	13.5	13.6	14.5	14.9	−11.2
New York	174	10.9	12.0	9.4	8.9	9.7	10.2	−11.0
Illinois	283	13.3	13.6	9.3	10.6	12.2	11.8	−8.3
North Carolina	176	12.1	11.2	10.3	11.0	11.4	11.2	−6.1
Arizona	156	13.2	13.0	9.5	9.4	12.5	11.5	−5.7
New Jersey	284	12.2	12.5	9.7	11.7	11.6	11.5	−4.6
Virginia	184	15.7	13.7	13.4	12.6	15.9	14.3	1.8
New York City	524	14.9	15.5	12.6	15.0	15.5	14.7	3.9
Florida	519	8.0	7.8	5.9	7.2	8.3	7.4	4.4
Washington	205	14.1	16.0	11.4	14.9	16.0	14.5	13.1
Georgia	255	13.3	15.2	11.4	12.3	16.2	13.7	21.8
**5-year average of 51–126 cases**								
Alaska	66	24.5	25.9	24.2	21.2	15.2	22.0	−38.1
Hawaii	104	39.7	31.5	27.8	32.2	33.0	32.8	−16.9
Alabama	81	17.8	23.2	14.5	17.8	16.1	17.8	−9.6
Nevada	60	9.1	6.7	6.7	7.6	8.3	7.7	−8.9
Indiana	108	18.4	18.6	14.7	19.0	17.7	17.7	−3.9
Tennessee	114	18.3	15.2	15.4	11.8	18.5	15.8	1.4
Colorado	59	9.0	10.2	7.4	8.2	9.1	8.8	1.5
Missouri	75	16.4	15.1	17.8	22.8	17.5	17.9	7.1
Wisconsin	51	11.2	12.6	7.5	18.8	12.2	12.5	8.6
Kentucky	64	21.2	23.3	18.5	20.9	23.7	21.5	11.5
Louisiana	95	18.8	16.9	14.3	16.6	21.1	17.6	12.2
Oklahoma	73	16.6	16.8	13.8	16.5	19.3	16.6	15.9
Oregon	74	12.2	13.5	12.5	14.1	14.3	13.3	17.6
Arkansas	67	16.3	12.9	13.6	19.9	20.6	16.6	26.2
Michigan	119	8.9	10.6	7.7	12.7	11.4	10.3	28.4
Mississippi	56	13.6	25.2	17.3	20.2	18.6	18.8	36.5
Connecticut	59	7.3	10.7	8.4	7.6	10.0	8.8	37.1
South Carolina	84	6.6	10.0	10.7	12.8	15.7	11.2	136.5
**5-year average of 1–50 cases**								
District of Columbia	22	28.3	19.9	19.9	12.1	12.1	18.6	−57.3
North Dakota	13	24.9	46.7	25.2	32.5	13.6	27.8	−45.6
Vermont	4	16.6	11.0	7.4	6.4	9.6	10.2	-42.4
Idaho	9	14.2	6.4	4.3	4.3	8.5	7.6	−39.6
Delaware	22	14.4	9.2	10.2	8.9	9.9	10.4	−31.1
New Mexico	33	14.2	13.7	9.6	7.8	11.4	11.3	−19.8
Rhode Island	15	11.0	8.5	4.9	8.7	9.9	8.6	−10.3
Nebraska	26	16.6	8.3	14.5	14.6	15.3	13.9	−7.8
New Hampshire	11	12.1	6.9	12.6	6.0	12.0	9.9	−0.8
Iowa	50	22.1	26.1	14.8	21.2	24.7	21.8	11.8
Maine	16	16.2	26.3	22.2	21.2	25.1	22.2	55.0
Kansas	39	10.1	12.3	13.3	13.6	16.5	13.2	62.9
South Dakota	13	13.5	33.7	22.4	30.0	26.2	25.3	93.6
Utah	25	4.9	6.6	8.5	5.4	9.7	7.0	98.0
West Virginia	9	3.8	3.6	25.4	13.6	17.0	12.8	350.9
Montana	4	0.0	9.5	4.7	0.0	8.5	4.4	—***

**Abbreviation:** TB = tuberculosis.

* Number of TB cases per 100,000 non-US-born persons. Population data from the Census Bureau’s American Community Survey, 2021. Jurisdictions were categorized into terciles based on the average number of cases during the 5-year reporting period for which the most recent data were available.

^†^ The 2025 national performance target for non-U.S.–born persons is 8.8 cases per 100,000 population. The 2025 National TB Program Objectives & Performance Targets reflect the projected performance of the top 10% of TB programs in the United States (https://www.cdc.gov/tb/education/pdf/2025_TB_Objectives.pdf).

^§^ The 2018–2022 national average incidence for non-U.S.–born persons was 13.1 cases per 100,000 population and was calculated as the 5-year sum of TB cases in all reporting areas divided by the 5-year sum of population in all reporting areas, multiplied by 100,000 (i.e., [sum of cases reported 2018 through 2022 ÷ sum of population 2018 through 2022] × 100,000).

^¶^ Reporting areas (i.e., jurisdictions) include all states, New York City, and the District of Columbia. These areas are included because they report TB surveillance data directly to CDC. Reporting areas are categorized into three groups based on the average number of TB cases reported over the 5-year period. The cutoffs for the groups were determined by the 5-year average number of cases that fell within the 33rd and 66th percentiles.

** Data for jurisdictions with <50 average cases should be interpreted with caution because of variability in performance results attributable to small denominators.

**^††^** Calculated as the sum of cases reported over the 5-year period divided by 5 (i.e., [sum of cases reported 2018 through 2022] ÷ 5).

**^§§^** Calculated as the sum of annual incidence divided by 5 (i.e., [sum of incidence reported 2018 through 2022] ÷ 5).

^¶¶^ Calculated as the difference in incidence between year 1 and year 5, divided by the incidence in year 1, multiplied by 100 (i.e., [(incidence in 2022 − incidence in 2018) ÷ incidence in 2018] × 100). Result is based on unrounded numbers.

*** Dash indicates that the relative change could not be calculated because no TB cases were reported among non–U.S.-born persons in 2018.

**TABLE 3:** Percentage of persons with tuberculosis disease who have initial drug-susceptibility test results reported,* by jurisdiction and reporting year — United States, 2018–2022^†,§^

Jurisdiction^¶,^**	5-year annual average number of cases^††^	% of cases					5-year annual average percentage^††^	Relative change from 2018 to 2022 (%)^§§^
		2018	2019	2020	2021	2022		
**5-year average ≥127 cases**								
Texas	1,048	88.4	93.0	96.9	96.2	93.7	93.6	6.0
New York City	524	97.8	96.3	97.8	97.7	99.3	97.8	1.5
Ohio	151	99.2	99.1	87.5	94.0	100.0	96.0	0.8
Minnesota	141	99.3	100.0	100.0	100.0	100.0	99.9	0.8
Arizona	156	99.3	99.2	99.1	100.0	100.0	99.5	0.7
Massachusetts	165	98.1	98.6	99.1	96.8	97.7	98.1	−0.4
North Carolina	176	98.2	98.2	100.0	99.3	97.7	98.7	−0.5
Virginia	184	100.0	100.0	99.2	100.0	99.3	99.7	−0.7
New Jersey	284	96.6	99.2	95.8	96.9	95.7	96.8	−1.0
Georgia	255	97.5	93.8	94.9	89.9	93.8	94.0	−3.9
California	1,901	98.4	97.7	97.7	96.3	94.3	96.9	−4.2
Washington	205	100.0	98.9	99.2	98.8	95.0	98.4	−5.0
Florida	519	98.4	97.3	98.6	96.4	92.4	96.6	−6.1
Maryland	183	98.8	95.3	98.3	95.6	91.2	95.8	−7.7
New York	174	95.4	99.3	100.0	99.2	85.4	95.9	−10.4
Pennsylvania	181	97.7	96.4	97.7	98.6	86.9	95.4	−11.1
Illinois	283	96.8	95.0	96.8	93.4	79.4	92.3	−18.0
**5-year average of 51–126 cases**								
Missouri	75	32.6	4.7	96.9	94.9	98.3	65.5	201.9
Hawaii	104	94.9	100.0	98.6	98.7	100.0	98.4	5.4
Indiana	108	97.7	98.6	97.2	100.0	100.0	98.7	2.4
South Carolina	84	98.6	98.5	100.0	100.0	100.0	99.4	1.4
Kentucky	64	98.0	97.5	100.0	100.0	98.0	98.7	0.1
Michigan	119	100.0	98.9	100.0	97.6	100.0	99.3	0.0
Colorado	59	100.0	100.0	97.1	100.0	100.0	99.4	0.0
Alaska	66	100.0	97.7	100.0	100.0	97.1	98.9	−2.9
Tennessee	114	98.9	96.9	96.5	96.4	95.8	96.9	−3.1
Nevada	60	98.2	100.0	100.0	100.0	94.4	98.5	−3.8
Alabama	81	97.3	96.0	86.7	95.1	93.2	93.7	−4.2
Louisiana	95	97.7	93.2	98.7	98.6	93.0	96.3	−4.8
Oregon	74	100.0	100.0	100.0	100.0	94.8	99.0	−5.2
Connecticut	59	100.0	98.2	100.0	100.0	94.1	98.5	−5.9
Mississippi	56	98.3	100.0	93.5	94.7	91.1	95.5	−7.3
Arkansas	67	94.9	100.0	97.9	96.2	85.7	94.9	−9.7
Oklahoma	73	100.0	98.3	95.9	91.7	88.5	94.9	−11.5
Wisconsin	51	97.7	100.0	100.0	96.4	0.0	78.8	−100.0
**5-year average of 1–50 cases**								
North Dakota	13	85.7	73.3	50.0	92.3	100.0	80.3	16.7
New Mexico	33	96.7	100.0	100.0	100.0	100.0	99.3	3.4
Utah	25	100.0	100.0	100.0	100.0	100.0	100.0	0.0
District of Columbia	22	100.0	100.0	100.0	100.0	100.0	100.0	0.0
Delaware	22	100.0	93.3	100.0	96.9	100.0	98.0	0.0
Maine	16	100.0	92.3	100.0	100.0	100.0	98.5	0.0
South Dakota	13	100.0	100.0	100.0	100.0	100.0	100.0	0.0
New Hampshire	11	100.0	100.0	91.7	100.0	100.0	98.3	0.0
Idaho	9	100.0	100.0	100.0	100.0	100.0	100.0	0.0
West Virginia	9	100.0	100.0	100.0	100.0	100.0	100.0	0.0
Vermont	4	100.0	100.0	100.0	100.0	100.0	100.0	0.0
Wyoming	1	100.0	100.0	—***	0.0	100.0	—	0.0
Rhode Island	15	91.7	88.9	100.0	91.7	90.0	92.4	−1.8
Iowa	50	100.0	100.0	100.0	100.0	97.7	99.5	−2.3
Kansas	39	100.0	100.0	94.1	100.0	86.4	96.1	−13.6
Nebraska	26	95.0	92.3	92.6	94.7	81.8	91.3	−13.9
Montana	4	100.0	100.0	100.0	100.0	60.0	92.0	−40.0

**Abbreviation:** TB = tuberculosis.

* Among persons with positive culture results. Jurisdictions were categorized into terciles based on the average number of cases during the 5-year reporting period for which the most recent data were available.

^†^ 2025 national performance target is 100%. The national performance targets reflect the projected performance of the top 10% of TB programs in the United States in 2025 (https://www.cdc.gov/tb/education/pdf/2025_TB_Objectives.pdf).

^§^ 2018–2022 national average was 97% and was calculated as the 5-year sum of persons in all reporting areas who had drug susceptibility test results divided by the 5-year sum of TB patients in all reporting areas who were culture positive, multiplied by 100 (i.e., [sum of patients with drug susceptibility test results 2018 through 2022 ÷ sum of patients with positive culture 2018 through 2022] × 100).

^¶^ Reporting areas (i.e., jurisdictions) include all states, New York City, and the District of Columbia. These areas are included because they report TB surveillance data directly to CDC. Reporting areas are categorized into three groups based on the average number of TB cases reported over the 5-year period. The cutoffs for the groups were determined by the 5-year average number of cases that fell within the 33rd and 66th percentiles.

** Data for jurisdictions with <50 average cases should be interpreted with caution because of variability in performance results attributable to small denominators.

^††^ Calculated as the sum of cases reported over the 5-year period divided by 5 (i.e., [sum of cases reported 2018 through 2022] ÷ 5).

^§§^ Calculated as the sum of annual percentages divided by 5 (i.e., [sum of percentages reported 2018 through 2022] ÷ 5).

^¶¶^ Calculated as the difference in percentage between year 1 and year 5, divided by the percentage in year 1, multiplied by 100 (i.e., [(percentage in 2022 minus percentage in 2018 ÷ percentage in 2018)] × 100). Result is based on unrounded numbers.

*** Dashes indicate that no patients met the indicator criteria for that year. Therefore, percentages or relative change could not be calculated.

**TABLE 4:** Percentage of contacts to sputum acid-fast bacillus smear-positive tuberculosis cases among persons who completed treatment for latent tuberculosis infection,* by jurisdiction and reporting year — United States, 2017–2021^†,§^

Jurisdiction^¶,^**	5-year annual average number of cases^††^	2017	2018	2019	2020	2021	5-year annual average percentage^§§^	Relative change from 2017 to 2021 (%)^¶¶^
**5-year average ≥131 cases**								
Texas	1,050	53.8	82.1	80.9	85.6	83.0	77.1	54.3
Illinois	290	73.5	76.3	93.3	87.5	93.1	84.7	26.7
Washington	196	76.6	79.2	81.5	85.6	96.3	83.8	25.7
New York	177	72.5	70.1	52.9	75.5	88.6	71.9	22.2
Arizona	163	74.3	86.9	76.0	77.2	83.8	79.6	12.8
Pennsylvania	185	82.4	78.7	94.4	88.2	90.6	86.8	10.0
New York City	539	82.3	79.2	79.7	79.7	88.5	81.9	7.5
North Carolina	186	82.9	79.8	79.5	81.0	87.7	82.2	5.8
California	1,943	70.4	58.3	69.1	73.8	73.7	69.1	4.7
Virginia	186	88.9	82.9	86.5	91.8	92.0	88.4	3.5
Florida	522	80.8	89.9	88.2	89.8	81.8	86.1	1.2
New Jersey	283	85.4	90.5	87.7	86.0	85.2	87.0	−0.2
Minnesota	150	83.7	77.2	77.3	78.6	83.3	80.0	−0.5
Ohio	152	86.2	90.9	90.9	77.8	82.9	85.7	−3.8
Georgia	261	78.1	91.5	81.3	74.1	67.6	78.5	−13.4
Massachusetts	176	61.8	84.0	70.8	68.4	40.0	65.0	−35.3
Maryland	193	73.2	78.8	77.0	84.5	42.4	71.2	−42.0
**5-year average of 50–130 cases**								
Connecticut	58	54.5	36.8	78.4	53.3	94.7	63.5	73.8
Kentucky	63	74.2	80.6	86.1	75.8	100.0	83.3	34.8
Arkansas	71	68.9	85.5	91.5	76.2	91.2	82.7	32.4
Louisiana	104	58.6	65.4	60.0	61.5	75.0	64.1	28.0
Mississippi	55	82.1	86.5	50.0	0.0	94.7	62.7	15.3
Wisconsin	50	73.3	90.0	100.0	52.9	83.3	79.9	13.6
Oregon	73	82.0	93.4	91.1	96.9	92.9	91.3	13.3
Hawaii	107	85.2	93.3	73.7	76.5	95.1	84.8	11.6
Oklahoma	67	82.8	78.1	78.3	87.2	90.5	83.4	9.3
Alaska	58	78.2	95.0	79.2	90.9	81.3	84.9	4.0
Indiana	109	90.0	92.2	82.7	91.4	91.7	89.6	1.9
Michigan	122	95.2	86.2	94.6	95.2	96.6	93.6	1.5
South Carolina	84	94.9	86.6	95.6	95.5	93.9	93.3	−1.1
Colorado	65	90.3	84.0	88.3	93.3	84.6	88.1	−6.3
Tennessee	118	91.4	80.0	93.9	88.3	80.0	86.7	−12.5
Nevada	64	93.5	82.5	88.1	90.3	78.4	86.6	−16.1
Alabama	92	90.6	94.6	88.6	53.6	74.1	80.3	−18.2
Missouri	79	—***	—	52.9	100.0	76.5	76.5	—
**5-year average of 1–49 cases**								
Maine	15	2.7	—	66.7	71.4	100.0	60.2	3,603.7
South Dakota	14	72.7	100.0	90.5	85.7	100.0	89.8	37.6
Utah	24	82.4	100.0	100.0	75.0	100.0	91.5	21.4
District of Columbia	27	90.0	75.0	71.4	100.0	100.0	87.3	11.1
New Hampshire	12	75.0	100.0	100.0	100.0	77.8	90.6	3.7
Iowa	47	84.6	62.3	72.4	64.7	85.2	73.8	0.7
Delaware	23	90.9	77.5	100.0	60.0	80.0	81.7	−12.0
New Mexico	34	90.9	96.2	100.0	100.0	66.7	90.8	−26.6
Rhode Island	14	83.3	41.7	100.0	100.0	60.0	77.0	−28.0
Nebraska	24	28.6	—	100.0	—	0.0	42.9	−100.0
North Dakota	14	75.0	100.0	100.0	85.7	—	90.2	−100.0
West Virginia	10	100.0	0.0	100.0	100.0	0.0	60.0	−100.0
Montana	3	100.0	—	0.0	0.0	0.0	25.0	−100.0
Idaho	9	0.0	100.0	0.0	0.0	100.0	40.0	NA
Vermont	4	0.0	0.0	—	—	0.0	0.0	NA
Wyoming	1	0.0	0.0	0.0	0.0	0.0	0.0	NA
Kansas	35	—	—	87.5	91.7	89.5	89.6	—

**Abbreviations:** AFB = acid-fast bacillus; LTBI = latent tuberculosis infection; NA = not applicable. TB = tuberculosis.

*Among contacts to sputum AFB smear-positive TB patients with newly diagnosed LTBI who started treatment for LTBI. Jurisdictions were categorized into terciles based on the average number of cases during the 5-year reporting period for which the most recent data were available.

^†^ 2025 national performance target is 93%. The national performance targets reflect the projected performance of the top 10% of TB programs in the United States in 2025 (https://www.cdc.gov/tb/education/pdf/2025_TB_Objectives.pdf).

^§^ 2017–2021 national average was 81.2% and was calculated as the 5-year sum of contacts to sputum AFB smear-positive TB patients with newly diagnosed LTBI in all reporting areas who completed treatment for LTBI divided by the 5-year sum of contacts to sputum AFB smear-positive TB patients with newly diagnosed LTBI in all reporting areas who started on treatment for LTBI, multiplied by 100 (i.e., [sum of newly diagnosed contacts who completed LTBI treatment 2017 through 2021 ÷ sum of newly diagnosed contacts who started LTBI treatment 2017 through 2021] × 100).

^¶^ Reporting areas (i.e., jurisdictions) include all states, New York City, and the District of Columbia. These areas are included because they report TB surveillance data directly to CDC. Reporting areas are categorized into three groups based on the average number of TB cases reported over the 5-year period. The cutoffs for the groups were determined by the 5-year average number of cases that fell within the 33rd and 66th percentiles.

** Data for jurisdictions with <50 average cases should be interpreted with caution because of variability in performance results attributable to small denominators.

^††^ Calculated as the sum of cases reported over the 5-year period divided by 5 (i.e., [sum of cases reported 2017 through 2021] ÷ 5).

^§§^ Calculated as the sum of annual percentages divided by 5 (i.e., [sum of percentages reported 2017 through 2021] ÷ 5).

^¶¶^ Calculated as the difference in percentage between year 1 and year 5, divided by the percentage in year 1, multiplied by 100 (i.e., [(percentage in 2021 minus percentage in 2017) ÷ percentage in 2017] × 100). Result is based on unrounded numbers.

*** Dashes indicate that no patients met the indicator criteria for that year. Therefore, percentages or relative change could not be calculated.

**TABLE 5:** Percentage of newly diagnosed tuberculosis cases among patients completing treatment ≤12 months,* jurisdiction and reporting year — United States, 2016–2020^†,§^

Jurisdiction^¶,^**	5-year annual average number of cases^††^	2016	2017	2018	2019	2020	5-year annual average percentage^§§^	Relative change from 2016 to 2020 (%)^¶¶^
**5-year average ≥131 cases**								
Pennsylvania	186	78.4	75.6	84.4	89.6	88.8	83.2	13.2
Ohio	150	77.1	83.7	86.6	86.5	86.1	84.2	11.6
Arizona	175	87.4	92.5	84.4	94.0	91.7	89.8	4.9
Washington	197	92.7	93.4	94.1	93.9	95.5	93.9	3.1
Georgia	277	88.9	86.3	92.8	90.0	91.3	89.7	2.7
Florida	550	93.1	95.2	97.2	97.4	95.6	95.6	2.7
California	2,005	88.4	90.2	88.4	88.5	90.0	89.1	1.8
Illinois	307	93.8	92.6	89.9	93.8	94.9	92.8	1.1
Virginia	194	92.4	97.2	94.0	93.8	92.4	94.0	−0.1
Massachusetts	184	88.5	94.6	83.9	85.2	87.9	88.1	−0.6
Texas	1,099	84.9	85.7	85.8	86.8	83.7	85.4	−1.5
New Jersey	284	93.8	92.9	92.1	93.2	92.2	92.9	−1.7
North Carolina	194	92.0	94.6	90.2	95.5	89.9	92.6	−2.2
New York	187	95.6	93.2	95.7	88.3	93.0	93.3	−2.7
Minnesota	157	94.2	95.7	92.1	89.5	91.4	92.8	−3.0
New York City	544	93.3	92.9	93.1	90.2	90.2	92.0	−3.3
Maryland	199	93.4	86.6	77.5	83.5	76.2	84.0	−18.4
**5-year average of 47–130 cases**								
Oregon	71	79.3	90.2	95.8	96.8	94.7	91.6	19.5
Arkansas	75	78.6	78.9	76.1	87.5	86.4	80.8	9.9
Mississippi	58	89.1	86.0	93.8	89.6	97.1	91.1	9.0
Nevada	63	95.3	98.5	100.0	97.6	100.0	98.4	4.9
Indiana	105	92.3	96.4	95.8	94.4	94.4	94.7	2.2
Louisiana	112	75.9	83.2	75.8	73.7	77.4	77.6	2.0
Colorado	66	89.1	91.2	96.6	94.5	90.0	92.5	1.0
South Carolina	87	94.0	92.0	93.4	95.0	94.3	93.6	0.4
Missouri	83	81.1	73.6	74.6	79.0	79.7	77.7	−1.8
Alaska	58	88.9	91.5	94.7	90.4	85.7	90.3	−3.6
Alabama	96	87.6	88.8	86.4	93.0	84.5	88.2	−3.6
Michigan	121	94.8	96.0	95.3	94.3	90.4	94.3	−4.7
Iowa	47	92.5	97.4	95.2	76.9	87.5	90.1	−5.4
Kentucky	70	96.1	98.0	85.1	96.0	88.7	93.1	−7.7
Hawaii	109	99.0	96.9	94.1	91.9	90.4	94.8	−8.7
Oklahoma	69	94.9	95.8	94.8	82.7	83.3	90.6	−12.2
Connecticut	57	97.6	98.0	100.0	92.5	85.0	94.7	−12.9
Tennessee	122	95.3	95.2	92.6	97.3	82.1	92.7	−13.9
**5-year average of 1–46 cases**								
Wisconsin	45	85.3	90.0	85.7	97.5	91.3	90.1	7.0
New Mexico	37	89.3	96.0	91.2	89.2	93.8	91.4	5.0
West Virginia	12	81.8	84.6	60.0	100.0	84.6	83.3	3.4
Kansas	34	91.9	100.0	100.0	100.0	93.1	96.3	1.3
Vermont	4	100.0	50.0	40.0	75.0	100.0	72.2	0.0
North Dakota	15	90.5	92.3	81.8	75.0	90.0	86.6	−0.5
Utah	25	94.4	95.8	100.0	95.5	91.7	95.0	−2.9
Delaware	18	100.0	100.0	87.5	87.5	92.3	92.6	−7.7
Idaho	12	93.3	88.9	75.0	100.0	85.7	88.9	−8.2
District of Columbia	28	94.7	76.7	92.6	86.4	85.7	86.6	−9.5
New Hampshire	13	100.0	88.2	100.0	100.0	88.9	94.2	−11.1
South Dakota	14	88.9	100.0	72.7	91.7	76.9	85.7	−13.5
Rhode Island	13	87.5	77.8	78.6	72.7	75.0	78.3	−14.3
Maine	17	95.2	100.0	84.6	87.5	78.6	89.0	−17.5
Nebraska	25	56.0	66.7	83.3	84.6	42.3	64.1	−24.5
Montana	4	100.0	100.0	100.0	0.0	33.3	78.6	−66.7
Wyoming	1	—***	100.0	100.0	100.0	—	100.0	—

**Abbreviation:** TB = tuberculosis.

* Jurisdictions were categorized into terciles based on the average number of cases during the 5-year reporting period for which the most recent data were available. Details for the calculation of completion of therapy within 12 months are published in Reported Tuberculosis in the United States, 2021 (https://www.cdc.gov/tb/statistics/reports/2021/table18.htm).

^†^ 2025 national performance target is 99%. The national performance targets reflect the projected performance of the top 10% of TB programs in the United States in 2025 (https://www.cdc.gov/tb/education/pdf/2025_TB_Objectives.pdf).

^§^ 2016–2020 national average was 89.7% and was calculated as the 5-year sum of TB patients in all reporting areas who completed treatment within 12 months divided by the 5-year sum of TB patients in all reporting areas who were eligible to complete treatment within 12 months, multiplied by 100 (i.e., [sum of patients who completed treatment 2016 through 2020 ÷ sum of patients eligible to complete treatment 2016 through 2020] × 100).

^¶^ Reporting areas (i.e., jurisdictions) include all states, New York City, and the District of Columbia. These areas are included because they report TB surveillance data directly to CDC. Reporting areas are categorized into three groups based on the average number of TB cases reported over the 5-year period. The cutoffs for the groups were determined by the 5-year average number of cases that fell within the 33rd and 66th percentiles.

** Data for jurisdictions with <50 average cases should be interpreted with caution because of variability in performance results attributable to small denominators.

^††^ Calculated as the sum of cases reported over the 5-year period divided by 5 (i.e., [sum of cases reported 2016 through 2020] ÷ 5).

^§§^ Calculated as the sum of annual percentages divided by 5 (i.e., [sum of percentages reported 2016 through 2020] ÷ 5).

^¶¶^ Calculated as the difference in percentage between year 1 and year 5, divided by the percentage in year 1, multiplied by 100 (i.e., [(percentage in 2020 minus percentage in 2016) ÷ percentage in 2016] × 100). Result is based on unrounded numbers.

*** Dashes indicate that there were no patients who met the indicator criteria for that year. Therefore, percentages or relative change could not be calculated.

### TB Incidence in the United States

The incidence of TB decreased in 37 of the 52 (71.2%) jurisdictions from 2018 to 2022 (Table 1). The national 5-year average incidence of TB was 2.5 cases per 100,000 persons. Overall, 15 (28.9%) jurisdictions had a 5-year average incidence that was at or below the national performance target of 1.3 (Figure); 28 jurisdictions (53.9%) had a 5-year average incidence that was at or below the national average of 2.5 but higher than 1.3. Programs in these 28 jurisdictions have not yet met the national performance target. Nearly all jurisdictions in T1 had an average 5-year incidence at or below the national average and performance target (16 of 17 [94.1%] jurisdictions) compared with 16 of 18 (88.9%) jurisdictions in T2 and 11 of 17 (64.7%) jurisdictions in T3.

**FIGURE:**
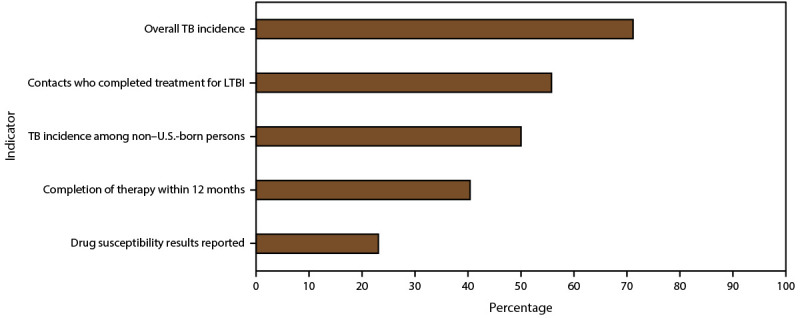
Percentage of tuberculosis programs with improved performance based on relative change* from year 1 to year 5 of the reporting period for which the most recent data were available for overall incidence,^†^ contacts who completed treatment for latent tuberculosis infection,^§^ tuberculosis incidence among non–U.S.-born persons,^¶^ completion of therapy within 12 months,^**^ and drug susceptibility results reported^††^ — United States **Source: **2005 National TB Program Objectives & Performance Targets (http://www.cdc.gov/tb/education/pdf/2025_TB_Objectives.pdf)**.** **Abbreviation:** TB = tuberculosis. * Relative change calculated as [(% from year 5 − % from year 1) ÷ % from year 1] × 100. ^†^ For the 5-year period from 2018 to 2022. Incidence calculated as number of TB cases per 100,000 persons (or number of TB cases among non–U.S.-born persons per 100,000 non–U.S.-born persons). Population data from Census Bureau’s American Community Survey, 2019. The 2025 national performance target for TB incidence for this indicator is 1.3 cases per 100,000 persons. ^§^ For the 5-year period from 2017 to 2021. Patients with latent TB infection completed treatment among contacts (to smear-positive TB cases) with diagnosed latent TB infection and started treatment. The 2025 national performance target for TB incidence for this indicator is 93%. ^¶^ For the 5-year period from 2018 to 2022. Incidence calculated as number of TB cases among non–U.S.-born persons per 100,000 non–U.S.-born persons. Population data from Census Bureau’s American Community Survey, 2019. The 2025 national performance target for TB incidence for this indicator is 8.8 cases per 100,000 persons. ** For the 5-year period from 2016 to 2020. Completion of treatment among patients eligible to complete treatment within 12 months. Details for the calculation of completion of therapy within 12 months are published in Reported Tuberculosis in the United States, 2021 (https://www.cdc.gov/tb/statistics/reports/2021/table18.htm). The 2025 national performance target for TB incidence for this indicator is 99%. ^††^ For the 5-year period from 2018 to 2022. Drug susceptibility test results among persons with positive culture results. For the 5-year period from 2018 to 2022. Drug susceptibility test results among persons with positive culture results. The 2025 national performance target for TB incidence for this indicator is 100%.

Although almost all jurisdictions in T1 had incidences that were below the national average or performance target, these jurisdictions also had the largest range in relative change in TB incidence for the 5-year period (−56.3% to 85.7%), an indication of the instability of data for jurisdictions with few TB cases; small changes in case counts can result in large percentage changes. In comparison, relative change ranged from −31.7% to 51.8% among jurisdictions in T2 and −26.0% to 28.6% in T3.

### TB Incidence Among Non–U.S.-Born Persons

Overall, eight of 51 (15.7%) jurisdictions (one state did not report any TB cases among non–U.S.-born persons during the 5-year period) had a 5-year average incidence that was at or below the national performance target of 8.8 cases per 100,000 non–U.S.-born persons (Figure); four of these eight jurisdictions were in T1 (Table 2). The incidence of TB among non–U.S.-born persons decreased in 26 of 51 (51.0%) jurisdictions from 2018 to 2022 (Table 2). Approximately two thirds (12 of 17 [70.6%]) of jurisdictions in T3 had decreases in the relative change in incidence among non–U.S.-born persons compared with approximately one third of jurisdictions in T2 (five of 18 [27.8%]) and over half in T1 (nine of 16 [56.3%]). Among jurisdictions in T3, eight of 17 (47.1%) had an average 5-year incidence at or below the national average of 13.1 compared with six of 18 (33.3%) in T2 and nine of 16 (56.3%) in T1. As with overall TB incidence, the largest range in relative change in TB incidence among non–U.S.-born persons from 2018 to 2022 occurred among jurisdictions in T1(−57.3% to 350.9%) compared with jurisdictions in T2 (−38.1% to 136.5%) and T3 (−33.0% to 21.8%).

### Initial Drug Susceptibility Test Results

The 5-year average percentages of persons having initial drug susceptibility test results reported during 2018–2022 in most jurisdictions (28 of 52, [53.9%]) met or exceeded the 5-year national average of 97% (Table 3). The 5-year average percentage could not be calculated for one jurisdiction in T1 because no cases with positive culture results were reported in 2020; therefore, no persons with diagnosed TB disease were eligible to be included in the indicator cohort for that year. The percentage that met or exceeded the national average was highest for jurisdictions in T1 (11 of 17 [64.7%]), followed by T2 (10 of 18 [55.6%]), and T3 (seven of 17 [41.2%]). The 2025 national performance target of 100% has been met in six jurisdictions, all of which were in T1; no jurisdictions in the other two categories met the 2025 national performance target.

Although half of the jurisdictions met or exceeded the national average for initial drug susceptibility results reported, only 12 of 52 (23.1%) had an increase in relative change from 2018 to 2022. In addition, 12 of 52 (23.1%) jurisdictions had no relative change in performance from 2018 to 2022; six of the 12 (50.0%) had a 5-year average performance that had already met the 2025 national performance target for reporting initial drug susceptibility test results.

### Contacts to Sputum AFB Smear-Positive TB Patients with a New Diagnosis of LTBI Who Completed Treatment

The relative change in the percentage of contacts who received a new diagnosis of LTBI who completed treatment increased in 29 of 52 (55.8%) jurisdictions from 2017 to 2021, signifying that, for most jurisdictions, steps have been taken to enhance performance for this indicator (Table 4). In addition, the same number of jurisdictions (although not the same jurisdictions) met or exceeded the national average of 81.2% for contacts who received a new diagnosis of LTBI and completed treatment (29 of 52 [55.8%]). However, only two jurisdictions have met or exceeded the 2025 national performance target of 93%. The percentage who met or exceeded the national average was highest among jurisdictions in T2 (12 of 18 [66.7%]), followed by jurisdictions in T3 (nine of 17 [52.9%]) and jurisdictions in T1 (eight of 17 [47.1%]).

Although the range of relative change in 5-year average performance was highest among jurisdictions in T1 (3,603.7% to −100.0%), these results are unreliable because of multiple jurisdictions with missing or incomplete contact investigation data (or incomplete data reporting), no contact investigation done, or no contacts receiving a diagnosis of LTBI. In contrast, jurisdictions in T2 and T3 had similar ranges in relative change (73.8% to −18.2% and 54.3% to −42.0%, respectively). This observation reinforces the importance of complete and accurate data collection and reporting for a better assessment of LTBI treatment outcomes.

### Completion of TB Therapy Within 12 Months

During 2016–2020, approximately two thirds (32 of 52 [61.5%]) of jurisdictions were at or above the national average of 89.7% for patients completing therapy within 12 months (Table 5). The percentage of jurisdictions with performance above the national average varied substantially between terciles: 11 of 17 (64.7%) in T3, 14 of 18 (77.8%) in T2, and seven of 17 (41.2%) in T1. Furthermore, 20 of the 52 jurisdictions (38.5%) had an increase in the percentage of patients completing therapy within 12 months from 2016 to 2020; this percentage is the second lowest performance improvement result among the indicators presented in this report (Figure). Performance improvement was similar between jurisdictions in T3 and T2 (eight of 17 [47.1%] and eight of 18 [44.4%], respectively). Jurisdictions in T1 had a slightly lower performance, with four of 17 (23.5%) jurisdictions with increased performance over the 5-year period. These results indicate that jurisdictions with a higher number of cases demonstrated more improvement for completion of treatment than jurisdictions with fewer cases.

Only one jurisdiction had 5-year average performance that met or exceeded the 2025 national performance target of 99% of patients completing TB therapy within 12 months. This jurisdiction (Wyoming) reported an average of one TB case per year over the 5-year period; therefore, comparing the performance of Wyoming with the performance in most other jurisdictions might not be useful.

Jurisdictions in T1 had the largest range of relative change in 5-year average performance (7.0% to − 66.7%). Jurisdictions in T2 and T3 had similar ranges of relative change (19.5% to −13.9% and 13.2% to −18.4%, respectively).

## Discussion

During 2018–2022, most TB programs had improvements in reducing overall TB incidence, and approximately half of programs had improvements in reducing incidence among non–U.S.-born persons. In addition, from 2017 to 2021, most programs increased the percentage of contacts receiving a diagnosis of LTBI who completed treatment. These results suggest that TB programs continue to strengthen activities that help identify persons with TB disease and LTBI and ensure they complete treatment in a timely manner (*16*). Improvements in reducing TB incidence do not correlate with improved performance across all indicators. Although a lower percentage of TB programs indicated improvement for initial drug susceptibility test results reported, this observation is partly because of the number of TB programs that had no relative change for this indicator because the programs were at 100% for the first and last year of the 5-year period, indicating a high level of performance overall.

TB programs are making progress in reducing TB incidence among non–U.S.-born persons; however, country of birth remains the most important risk factor for TB (*17*), emphasizing the need for ongoing efforts to reduce incidence of TB among non–U.S.-born persons. Furthermore, reactivation of LTBI is the most common cause of development of TB among non−U.S.-born persons. As a result, the diagnosis and treatment of LTBI play a vital role in TB elimination (*17*). Although this report provides insight into program performance for completion of therapy among contacts who received a diagnosis of LTBI, TB programs would benefit from additional information (e.g., treatment status and country of birth) about all persons diagnosed with LTBI, not just newly diagnosed contacts.

The low percentage of TB programs indicating improved performance for completion of therapy within 12 months from 2016 to 2020 might be attributed to multiple factors. First, TB treatment is a long and often resource-intensive process that can be affected by various factors (e.g., drug resistance, comorbidities, and socioeconomic factors) (*18*). Persons with more complicated TB cases, including drug resistance, drug intolerance, severe disease, or other concurrent illnesses or conditions, might require longer and more complex treatment regimens. As a result, the presence of a substantial number of such cases within TB programs might have contributed to the lower overall percentage of improvement. Second, the 5-year reporting period for completion of therapy in this report overlaps with the COVID-19 pandemic, which impacted TB diagnosis and management in a wide range of ways. The implementation of social distancing measures, quarantine protocols, and the diversion of health care resources to manage COVID-19 cases were experienced by most TB programs (*19*). These disruptions might have affected the ability of staff members in certain TB programs to implement directly observed therapy, which is the standard of care to ensure treatment completion (*18*).

TB programs whose jurisdictions were in T1 over a 5-year period tended to have higher performance improvement (measured by a decrease in relative change for incidence [e.g., TB incidence] and an increase in relative change for indicators measured by percentages [e.g., initial drug susceptibility test results]) than TB programs whose jurisdictions were in T2 or T3 with higher numbers of TB cases. This performance is likely because of small absolute changes associated with a lower number of TB cases leading to larger percentage changes.

The results presented in this report use the 2025 national performance targets as a measure of TB program effectiveness. Although certain TB programs have already achieved the 2025 performance target for specific indicators, these targets are designed to be aspirational and are not mandatory benchmarks for all TB programs (*14*). The targets should be interpreted as long-term objectives and should be used alongside other metrics (e.g., the national average) to provide a more comprehensive assessment of TB program performance.

Although this report provides a comparison of TB program performance on these five indicators, the report is not intended to penalize or negatively affect any TB programs or staff members or be used in a competitive manner. Rather, TB programs can use this report to monitor progress of TB prevention and control activities and determine where improvements can be made. Because this is the only report that allows TB programs to review the trend performance of other programs, it can provide context for where to focus improvement efforts based on successes of other TB programs or, conversely, for TB programs to understand where they have been successful compared with other programs. In addition, this report can facilitate communication between TB programs and generate ideas for sharing and learning in regional or national meetings, conferences, and trainings.

## Limitations

The findings in this report are subject to at least five limitations. First, TB program performance involves programmatic and contextual factors (e.g., population demographics and complexity of TB cases) that are not measured by indicator data. Second, TB programs vary in size and resources and, although these differences were accounted for by grouping based on the number of TB cases reported, the variability of case counts within each group is still large; therefore, not all TB programs within each group are comparable. Third, other ways exist to assess TB programs with similar characteristics (e.g., by age, race, or other demographic characteristics of persons with TB disease; by the cohort or denominator of persons who meet specific indicator criteria; or by geography) and differences that affect program performance are not only based on the number of persons with TB in the associated jurisdiction. However, this study does not examine alternative methods for grouping TB programs. Fourth, the relative change calculation is subject to the first and last year of the 5-year period and does not account for variability within that period. Finally, for TB programs that report few cases, a small absolute change can lead to a large percentage change that can be misleading and difficult to interpret. Therefore, results for TB programs with 1–50 cases should be interpreted with caution.

## Future Direction

To sustain progress toward TB elimination, maintaining strong evaluation and performance monitoring systems is important. Continued support for NTIP and regular assessment and improvement of indicators are essential to adapt to evolving TB trends and challenges. In addition, TB programs might benefit from a greater exchange of information and best practices, enabling them to learn from each other's successes and challenges. Data from NTIP can also play an important role in resource allocation and garnering support for TB control; TB programs can use this information to guide public health policy decisions at both the state and Federal levels. Finally, implementing comprehensive LTBI surveillance that includes data on the country of birth would enable establishment of specific objectives, performance indicators, and targets stratified by U.S.-born and non–U.S.-born persons. This approach would provide TB programs with the means to effectively monitor performance in diagnosing and treating persons with LTBI.

## Conclusion

The findings in this report indicate that most TB programs have indicated improvement in reducing TB incidence and increasing the percentage of contacts with LTBI who complete treatment. These activities are critical in preventing the spread of TB. Continuous monitoring and evaluation, along with subsequent programmatic actions aimed at enhancing performance in identified areas, will be essential for improving strategies to decrease the incidence of TB.
